# Urban and agricultural soils in Southern California are a reservoir of carbapenem‐resistant bacteria

**DOI:** 10.1002/mbo3.1034

**Published:** 2020-04-03

**Authors:** Nicolas V. Lopez, Cameron J. Farsar, Dana E. Harmon, Cristian Ruiz

**Affiliations:** ^1^ Department of Biology California State University Northridge Northridge CA USA

**Keywords:** *Achromobacter*, *Bradyrhizobium*, carbapenemase, carbapenem‐resistant bacteria, *Cupriavidus*, *Enterococcus*, *Planomicrobium*, *Pseudomonas*, soil, *Stenotrophomonas*

## Abstract

Carbapenems are last‐resort β‐lactam antibiotics used in healthcare facilities to treat multidrug‐resistant infections. Thus, most studies on identifying and characterizing carbapenem‐resistant bacteria (CRB) have focused on clinical settings. Relatively, little is still known about the distribution and characteristics of CRBs in the environment, and the role of soil as a potential reservoir of CRB in the United States remains unknown. Here, we have surveyed 11 soil samples from 9 different urban or agricultural locations in the Los Angeles–Southern California area to determine the prevalence and characteristics of CRB in these soils. All samples tested contained CRB with a frequency of <10 to 1.3 × 10^4^ cfu per gram of soil, with most agricultural soil samples having a much higher relative frequency of CRB than urban soil samples. Identification and characterization of 40 CRB from these soil samples revealed that most of them were members of the genera *Cupriavidus*, *Pseudomonas,* and *Stenotrophomonas.* Other less prevalent genera identified among our isolated CRB, especially from agricultural soils, included the genera *Enterococcus*, *Bradyrhizobium*, *Achromobacter,* and *Planomicrobium*. Interestingly, all of these carbapenem‐resistant isolates were also intermediate or resistant to at least 1 noncarbapenem antibiotic. Further characterization of our isolated CRB revealed that 11 *Stenotrophomonas*, 3 *Pseudomonas*, 1 *Enterococcus*, and 1 *Bradyrhizobium* isolates were carbapenemase producers. Our findings show for the first time that both urban and agricultural soils in Southern California are an underappreciated reservoir of bacteria resistant to carbapenems and other antibiotics, including carbapenemase‐producing CRB.

## Graphical abstract

The study reported here is the first specific study about the distribution and characteristics of carbapenem‐resistant bacteria (CRB) in urban and agricultural environmental soils in the United States. We have found that the soils from Los Angeles–Southern California area are an underappreciated reservoir of bacteria resistant to carbapenems and other antibiotics, including carbapenemase‐producing CRB. Our findings also show a much higher relative frequency of CRB on soils from locations adjacent to farms compared to soils from urban locations.

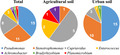

## 1 INTRODUCTION

Carbapenems are broad‐spectrum β‐lactam antibiotics that act as potent inhibitors of bacterial cell wall synthesis because of their high affinity for penicillin‐binding proteins (Papp‐Wallace, Endimiani, Taracila, & Bonomo, 2011). While most β‐lactams have a cishydroxyethyl side chain, carbapenems have a transhydroxyethyl side chain. This unique feature confers carbapenems increased resistance to hydrolysis by most β‐lactamases, including extended‐spectrum β‐lactamases, and thus has led to their use as last‐resort drugs to treat multidrug‐resistant infections (Papp‐Wallace et al., 2011; Vardakas, Tansarli, Rafailidis, & Falagas, 2012).

Carbapenem‐resistant bacteria (CRB), especially Enterobacteriaceae, *Pseudomonas aeruginosa,* and *Acinetobacter baumanii*, have been designated by the Centers for Disease Control and Prevention (CDC) and other health organizations as a major public health threat because the infections they cause are difficult to treat, their high associated mortality rates, and their rising prevalence in healthcare settings (Centers for Disease Control & Prevention, 2013a, 2013b; Cuzon et al., 2011; Guh et al., 2015).

Resistance to carbapenems can occur through three major mechanisms: decreased outer membrane permeability (Livermore, Mushtaq, & Warner, 2005; Shin et al., 2012; Warner et al., 2013), increased efflux (Livermore et al., 2005; Papp‐Wallace et al., 2011; Rodríguez‐Martínez, Poirel, & Nordmann, 2009; Warner et al., 2013), and production of carbapenemases, which are unique β‐lactamases capable of degrading carbapenems (Marsik & Nambiar, 2011; Queenan & Bush, 2007). Carbapenemase‐producing CRB (CP‐CRB) are especially concerning because carbapenemase genes are often located on transmissible genetic elements that can quickly spread to other bacteria (Mathers et al., 2011; Walsh, 2010).

Because the use of carbapenems is restricted to healthcare facilities (Bradley et al., 1999; Paterson, 2000), most studies on isolating and characterizing CRB have also focused on these and immediately related settings (Gupta, Limbago, Patel, & Kallen, 2011; Kallen, Hidron, Patel, & Srinivasan, 2010; Khuntayaporn, Montakantikul, Mootsikapun, Thamlikitkul, & Chomnawang, 2012; Rhomberg & Jones, 2009; Ssekatawa, Byarugaba, Wampande, & Ejobi, 2018). However, other β‐lactams including extended‐spectrum penicillins and cephalosporins are used to treat patients outside healthcare facilities and are used in agriculture as well. For example, in the United States, penicillins account for 12% of antibiotics used in food‐producing animals (United States Food & Drug Administration Center for Veterinary Medicine, 2017). Even though there is no established relationship between the broad use of β‐lactams or extended‐spectrum β‐lactams and resistance to carbapenems, the use of these and other drugs is predicted to cause selection favoring carbapenem resistance in the environment (Meletis, 2016; Mollenkopf et al., 2017). Recent findings of CRB in environmental samples from Europe, Africa, Asia, and North America (Adelowo, Vollmers, Mäusezahl, Kaster, & Müller, 2018; Ash, Mauck, & Morgan, 2002; Aubron, Poirel, Ash, & Nordmann, 2005; Di, Jang, Unno, & Hur, 2017; Girlich, Poirel, & Nordmann, 2010; Harmon et al., 2019; Henriques et al., 2012; Hrenovic et al., 2019; Isozumi et al., 2012; Mills & Lee, 2019; Poirel et al., 2012; Potron, Poirel, Bussy, & Nordmann, 2011; Sivalingam, Pote, & Prabakar, 2019; Tacão, Correia, & Henriques, 2015; Zou et al., 2020; Zurfluh, Hachler, Nuesch‐Inderbinen, & Stephan, 2013) seem to support this hypothesis. However, further studies are needed to fully understand the role of the environment as a reservoir of CRB and carbapenem resistance genes.

Knowledge about the environmental distribution and characteristics of CRB is especially lacking in the United States. For example, there have only been three studies about CRB in freshwater environments in the United States (Ash et al., 2002; Aubron et al., 2005; Harmon et al., 2019) and no specific studies about the prevalence or characteristics of CRB in U.S. soils. However, recent studies in soil and related environmental samples from Africa and Europe suggest that soil may be an underappreciated reservoir of CRB. For example, CRB including CP‐CRB have been isolated from agricultural and nonagricultural soil samples from Algeria, Spain, England, Germany, Denmark, and Norway (Gudeta et al., 2016) and Croatia (Hrenovic et al., 2019), as well as from swine and poultry farms from Germany (Borowiak et al., 2017; Fischer et al., 2013), and natural soil samples from Algeria (Djenadi, Zhang, Murray, & Gaze, 2018), among other locations.

Although there are no specific studies about the prevalence or characteristics of CRB in U.S. soils, a few studies suggest that CRB may also be prevalent in U.S. soils. For example, a study on soil samples from the Midwestern United States that used penicillins as selective agents identified three isolates that were carbapenem‐resistant (Crofts et al., 2018). CRB and CP‐CRB have also been isolated from fecal samples from dairy farms in New Mexico and Texas (Webb et al., 2016), as well as from fecal and environmental samples recovered from a swine nursery in Ohio (Mollenkopf et al., 2017). These findings are very significant because farm animal feces are routinely used as manure, which may lead to the spread of CRB and carbapenemase genes to the soil, water, and other environments.

To contribute to addressing the information gap about the role of U.S. soils as potential sinks and sources of CRB, we report here the first study specifically aimed at determining the prevalence and characteristics of CRB in soil from the West Coast of the United States. Our findings indicate that both urban and agricultural soils from the highly populated Los Angeles–Southern California area are a significant reservoir of CRB and CP‐CRB, which we found to be also resistant to other classes of antibiotics as well.

## 2 MATERIALS AND METHODS

### 2.1 Collection of soil samples and isolation of carbapenem‐resistant bacteria

**FIGURE 1:**
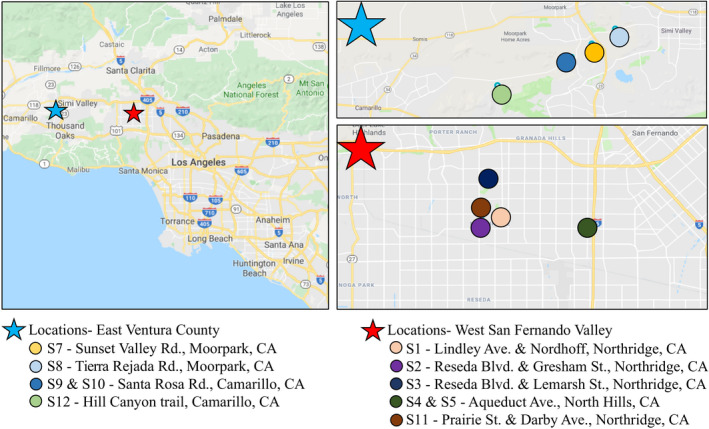
Map of the location of the soil samples analyzed in this study. Left panel: A general map of the Southern California region with the two major areas sampled in the East Ventura County (labeled with a blue star) and the West San Fernando Valley County (labeled with a red star). Top right panel: Detailed map of the soil locations sampled in the East Ventura County. Bottom right panel: Detailed map of the soil locations sampled in the West San Fernando Valley County

We collected 11 different soil samples from 9 different locations in the Los Angeles (California) area between June 2016 and January 2019. The location (Figure 1) and characteristics of sampling sites are summarized in Table 1. For each sample, we collected surface soil in 50‐ml sterile conical tubes and immediately transported the sample to the laboratory. We then weighed 4 g of the soil sample into a sterile 15‐ml conical tube, added 10 ml of sterile saline (0.85% NaCl), and vortexed the mixture continuously for 5 min to homogenize the sample and extract the bacteria present in the soil. Soil debris was then removed by centrifugation for 10 min at 1,000 × *g*, and the supernatant containing the extracted soil bacteria collected for subsequent analyses.

**TABLE 1:** Summary of the origin, count of total gram‐negative bacteria, and count of carbapenem‐resistant bacteria (CRB) obtained for the 11 soil samples from the Los Angeles–Southern California area tested in this study

Sample	Date	Location (Type)	Urban/agricultural	GPS location	Total bacteria (cfu/g)	CRB (cfu/g)
S1	10/3/2016	Lindley Ave. and Nordhoff St., Northridge (adjacent to CSUN Pond)	Urban	34.235587–118.5274932	1.2·10^5^	8.5·10^2^
S2	1/9/2017	Reseda Blvd. and Gresham St., Northridge (highly transited intersection)	Urban	34.2307707–118.5382339	9.6·10^4^	<10
S3	2/4/2017	Reseda Blvd. and Lemarsh St., Northridge (Northridge Recreation Center park)	Urban	34.2543024–118.5345628	TNTC	<10
S4	4/16/2017	Aqueduct Ave., North Hills (private chicken coop, sample A)	Urban	34.2308032–118.4751102	3.0·10^4^	1.3·10^4^
S5	4/16/2017	Aqueduct Ave., North Hills (private chicken coop, sample B)	Urban	34.2308032–118.4751102	3.0·10^4^	1.3·10^4^
S7	8/6/2017	Sunset Valley Rd., Moorpark (adjacent to produce farm)	Agricultural	34.2558565–118.8558643	1.9·10^3^	1.6·10^3^
S8	8/6/2017	Tierra Rejada Rd., Moorpark (adjacent to horse farm)	Agricultural	34.26555732–118.8345638	2.7·10^3^	5.6·10^2^
S9	8/6/2017	Santa Rosa Rd. and Moorpark Rd., Camarillo (adjacent to an avocado orchard, sample A)	Agricultural	34.2461891–118.8708311	1.1·10^3^	2.8·10^2^
S10	8/6/2017	Santa Rosa Rd. and Moorpark Rd., Camarillo (adjacent to an avocado orchard, sample B)	Agricultural	34.2461891–118.8708311	2.4·10^3^	4.4·10^2^
S11	12/26/2018	Prairie Rd. and Darby Ave., Northridge (grass area recently fertilized)	Urban	34.2391393–118.5360182	1.3·10^5^	2.5·10^2^
S12	1/7/2019	Hill Canyon trail, Camarillo (hiking trail near a strawberry farm)	Agricultural	34.2281655–118.9322636	9.3·10^4^	3.0·10^2^

Abbreviation: TNTC, too numerous to count.

The total count of bacteria was determined using MacConkey medium (Fisher Scientific) as a primary selection for enteric bacteria and gram‐negatives, which were the main target in our study. The bacterial count was determined by direct plating of 100 µl of soil supernatant as well as by spot plating of 10 µl of a 10^0^ to 10^–4^ dilution bank of soil supernatants in sterile saline on MacConkey agar plates, followed by incubation for 24 hr at 37°C. The count of carbapenem‐resistant bacteria (CRB) was determined by the same procedure except for using MacConkey agar plates containing 4 µg/ml of meropenem (Ark Pharm, Inc.), which is the Clinical Laboratory Standards Institute (CLSI) minimum inhibitory concentration (MIC) clinical breakpoint for this antibiotic in Enterobacteriaceae (Clinical & Laboratory Standards Institute, 2018). We selected meropenem because it is the most commonly prescribed carbapenem in the United States and is highly active against a broad spectrum of gram‐negative bacteria (Papp‐Wallace et al., 2011). Because of the low concentration of CRB in samples S2 and S3, all 10 ml of supernatant containing the extracted soil bacteria were concentrated by filtration using 0.45‐µm filters (Merck Millipore). The filters were then placed onto MacConkey‐meropenem plates as described above to obtain CRB colonies.

For each sample, we patched up to 50 distinct meropenem‐resistant colonies on Mueller‐Hinton (Fisher Scientific) agar plates supplemented with meropenem at 4 μg/ml (Enterobacteriaceae breakpoint) and 16 μg/ml (CLSI meropenem MIC breakpoint for other non‐Enterobacteriaceae gram‐negatives; Clinical & Laboratory Standards Institute, 2018). Growth in at least 4 μg/ml of meropenem was confirmed for nearly all patched colonies. In total, we selected 40 CRB isolates—up to 8 distinct CRB isolates per sample, prioritizing those that grew in 16 μg/ml of meropenem—for culturing, long‐term storage at −80°C, and preparation of cell suspension templates for PCR, as previously described (Harmon et al., 2019).

### 2.2 Identification of CRB by PCR and sequencing of the 16S rRNA gene, and oxidase test

The 40 selected soil CRB isolates were identified following the procedures described in Harmon et al. (2019). Briefly, we used PCR amplification of the 16S rRNA gene of each selected isolate, followed by Sanger sequencing, BLAST analysis (Altschul et al., 1997) of the obtained sequences, and oxidase test analysis. The oxidase test was used to further distinguish between closely related *S. maltophilia*, which is oxidase negative, and *Pseudomonas* species, most of which are oxidase‐positive (Bergey & Holt, 1994).

Besides, we constructed a phylogenetic tree for each genus isolated in our study (*Achromobacter, Bradyrhizobium, Cupriavidus, Enterococcus, Planomicrobium, Pseudomonas,* and *Stenotrophomonas*) to further characterize the taxonomic relationship between our soil isolates across different locations, as well as between our isolates and isolates from previous studies. We used MEGA X 10.1 software (Hall, 2013) to align the 16S rRNA genes and construct phylogenetic trees based on the Jukes–Cantor model and the neighbor joining method.

### 2.3 Determination of the antibiotic susceptibility profile of the isolated CRB

Determination of the antibiotic susceptibility profile of the 40 selected carbapenem‐resistant isolates was performed using the CLSI disk diffusion method (Clinical & Laboratory Standards Institute, 2018) and the reference strain *Escherichia coli* ATCC 25922 as quality control, as previously described (Harmon et al., 2019). The meropenem, imipenem, cefotaxime, ciprofloxacin, gentamicin, and tetracycline antibiotic disks were purchased from Becton Dickinson. To determine whether an isolate was susceptible, intermediate, or resistant to an antibiotic, we used CLSI zone diameter breakpoint values (Clinical & Laboratory Standards Institute, 2018). Unless otherwise indicated, for taxa in which the CLSI zone diameter breakpoints are not provided, we used the CLSI Enterobacteriaceae breakpoint values (Clinical & Laboratory Standards Institute, 2018).

### 2.4 Identification of carbapenemase‐producing isolates by the CarbaNP and mCIM assays, and detection of the L1 carbapenemase gene in *Stenotrophomonas* isolates

We identified carbapenemase‐producing CRB isolates using the CarbaNP assay (Dortet, Poirel, & Nordmann, 2012a, 2012b; Nordmann, Poirel, & Dortet, 2012). The assay was performed as described by CLSI (Clinical & Laboratory Standards Institute, 2018) using 6 mg/ml or either meropenem or imipenem. For each CRB isolate, colonies were grown overnight on plain Mueller‐Hinton agar (to detect constitutively expressed carbapenemases) and Mueller‐Hinton agar with the highest concentration of meropenem with growth (to detect inducible carbapenemases). Isolates that turned yellow at 37°C within 2 hr in the presence of meropenem or imipenem were considered carbapenemase‐positive. Isolates that were positive for carbapenemase production when grown on Mueller‐Hinton agar with the antibiotic but negative when grown on plain Mueller‐Hinton were considered to have an inducible carbapenemase.

For CarbaNP‐positive isolates, we confirmed that they produce carbapenemases by the modified Carbapenem Inactivation Method (mCIM; Pierce et al., 2017). This assay was performed as described by CLSI (Clinical & Laboratory Standards Institute, 2018). A zone of inhibition between 6 and 15 mm for *E. coli* ATCC 25922 when grown in the presence of a meropenem disk previously incubated in the presence of the isolate to be tested was a confirmed carbapenemase‐positive isolate.

PCR amplification to confirm the presence of the L1 carbapenemase gene (*bla*_L1_) in carbapenemase‐producing *Stenotrophomonas* isolates was performed using the primers and program described by Henriques et al. (2012) to amplify *bla*_L1_ as previously described (Harmon et al., 2019).

## 3 RESULTS

### 3.1 Distribution, frequency, and identification of carbapenem‐resistant bacteria in soil samples from the Los Angeles–Southern California area

We analyzed 11 different soil samples from 9 different urban and agricultural locations in the Los Angeles–Southern California area (United States; Figure 1; Table 1). Using meropenem as a selective agent, we found that all soil samples analyzed contained CRB. The frequency of CRB in these samples was between <10 and 1.3 × 10^4^ cfu per gram of soil (Table 1). Interestingly, S4 and S5, the two samples with the most abundance of CRB, were obtained from the soil of a private urban chicken coop, which suggests that animal feces might be an important contributor to soil CRB. Overall, samples could be classified into those with a low relative frequency of CRB (<1%) compared to the total bacterial counts obtained (S1–S3 and S11–S12; mostly urban soils) and those with a high relative frequency of CRB (18%–80%, urban chicken coop, and most agricultural soil samples) compared to the total bacterial count obtained (S4–S10; Table 2).

**TABLE 2:** Summary of the number and characteristics of soil carbapenem‐resistant bacteria isolated from samples described in Table 1

Genus	Sample of origin	Number of isolates	Number of CP^a^ isolates	Antibiotic resistant/intermediate (number of isolates)^b^
*Achromobacter*	S10	1	0	MP (1), CF (1)
*Bradyrhizobium*	S11	1	1	MP (1), IM (1), CF (1), CI (1), GE (1), TE (1)
*Cupriavidus*	S2, 7, 8, 9	8	0	MP (8), IM (2), CF (2), GE (4)
*Enterococcus*	S7, 11, 12	3	1	MP (3), IM (2), CF (3), GE (1)
*Planomicrobium*	S7	1	0	MP (1), IM (1), CF (1), GE (1), TE (1)
*Pseudomonas*	S2, 3, 4, 5, 11	15	3	MP (15), IM (5), CF (14), GE (1), TE (1)
*Stenotrophomonas*	S1, 7, 11	11	11	MP (11), IM (11), CF (3), GE (8), TE (8)
Total		40	16	MP (40), IM (22), CF (33), CI (1), GE (17), TE (11)

^a^ CP = carbapenemase‐producing isolates as determined by the CarbaNP test and confirmed using the mCIM method.

^b^ The number of isolates that were resistant or intermediate to meropenem (MP), imipenem (IM), cefotaxime (CF), ciprofloxacin (CI), gentamicin (GE), and tetracycline (TE) is shown in parentheses. The detailed antibiotic susceptibility profile and carbapenemase production result for each isolate are provided in Table 3.

**FIGURE 2:**
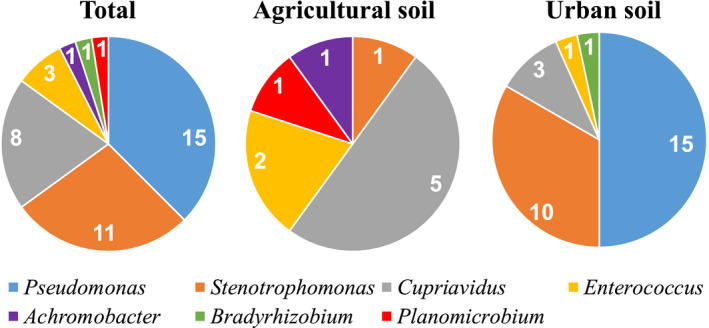
The abundance of the seven genera of carbapenem‐resistant isolates from soil identified in this study: Total abundance is shown on the left chart, abundance in agricultural soils is shown in the center chart, and abundance in urban soils is shown on the right chart

**FIGURE A1:**
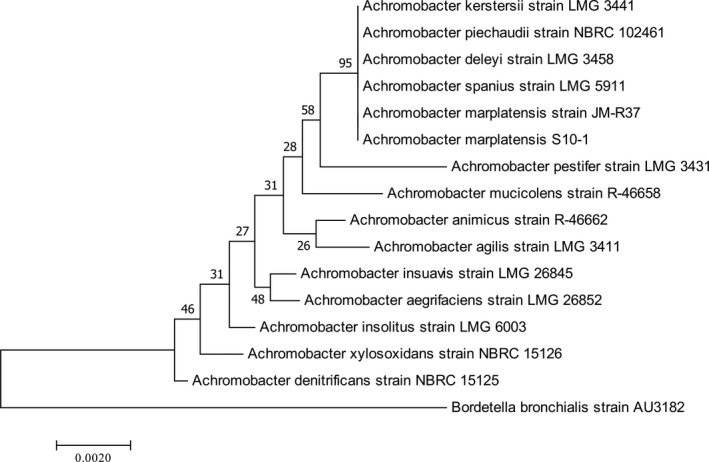
Phylogenetic tree constructed using 16S rRNA gene sequences showing the relatedness between the *Achromobacter* isolate from this study and *Achromobacter* isolates from previous studies. The scale bar at the bottom represents the number of nucleotide substitutions per site

**FIGURE A2:**
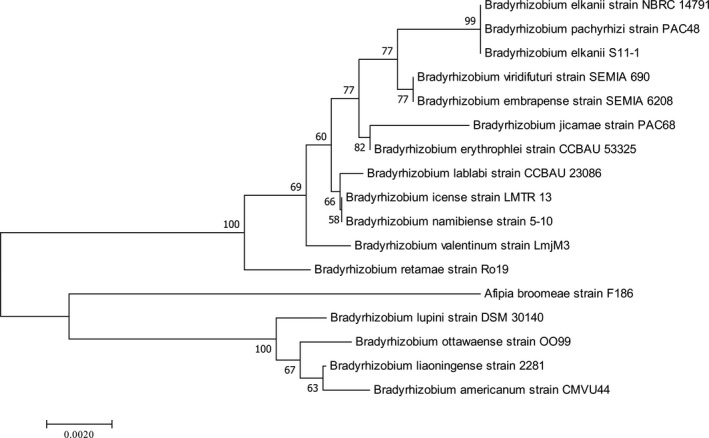
Phylogenetic tree constructed using 16S rRNA gene sequences showing the relatedness between the *Bradyrhizobium* isolate from this study and *Bradyrhizobium* isolates from previous studies. The scale bar at the bottom represents the number of nucleotide substitutions per site

**FIGURE A3:**
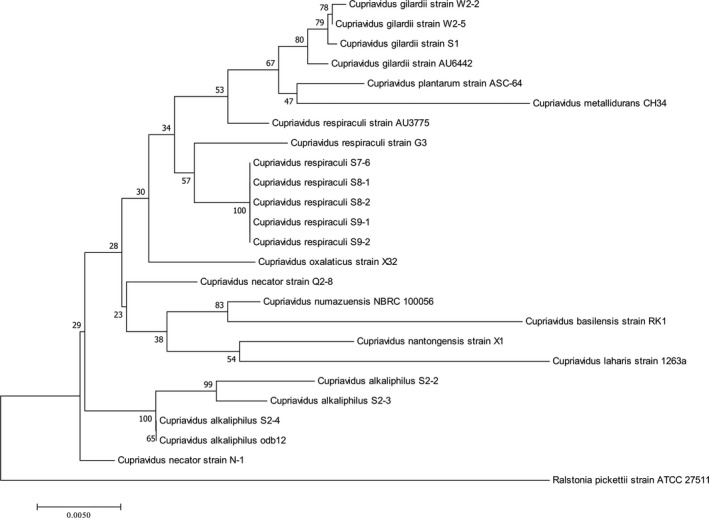
Phylogenetic tree constructed using 16S rRNA gene sequences showing the relatedness between *Cupriavidus* isolates from this and previous studies. The scale bar at the bottom represents the number of nucleotide substitutions per site

**FIGURE A4:**
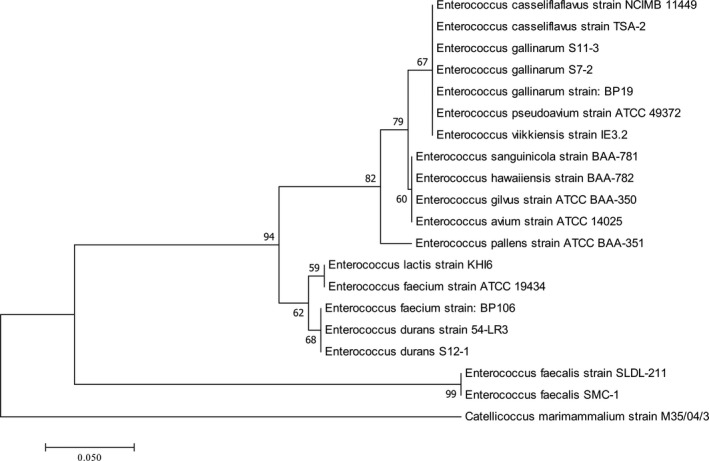
Phylogenetic tree constructed using 16S rRNA gene sequences showing the relatedness between *Enterococcus* isolates from this and previous studies. The scale bar at the bottom represents the number of nucleotide substitutions per site

**FIGURE A5:**
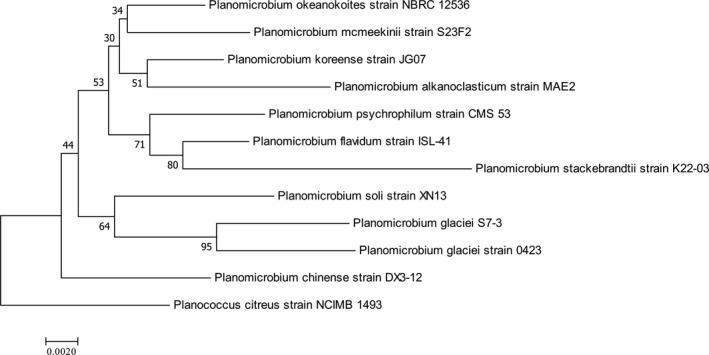
Phylogenetic tree constructed using 16S rRNA gene sequences showing the relatedness between the *Planomicrobium* isolate from this study and *Planomicrobium* isolates from previous studies. The scale bar at the bottom represents the number of nucleotide substitutions per site

**FIGURE A6:**
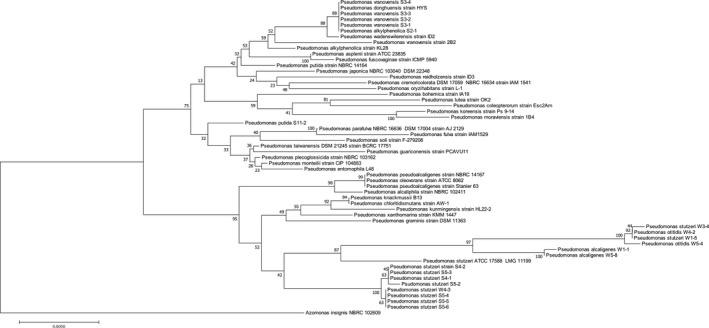
Phylogenetic tree constructed using 16S rRNA gene sequences showing the relatedness between *Pseudomonas* isolates from this and previous studies. The scale bar at the bottom represents the number of nucleotide substitutions per site

**FIGURE A7:**
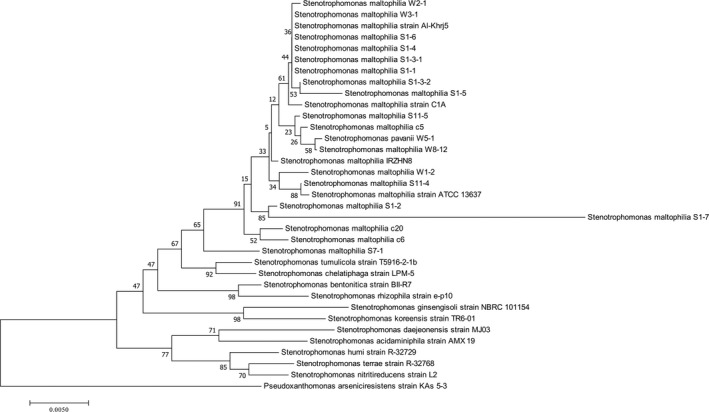
Phylogenetic tree constructed using 16S rRNA gene sequences showing the relatedness between *Stenotrophomonas* isolates from this and previous studies. The scale bar at the bottom represents the number of nucleotide substitutions per site

We selected a total of 40 CRB isolates for further identification and characterization. We identified them using their 16S rRNA gene sequence as well as phylogenetic analyses (Figure 2 and Figures A1, A2, A3, A4, A5, A6, A7). We also used the oxidase test to distinguish between members of the *Stenotrophomonas* genus and closely related members of the genus *Pseudomonas*. We preliminarily identified our isolates as 1 *Achromobacter marplatensis*, 1 *Bradyrhizobium elkanii,* 8 *Cupriavidus* (3 *C. alkaliphilus* and 5 *C. respiraculi*), 3 *Enterococcus (1 E. durans and 2 E. gallinarum), 1 Planomicrobium glaciei,* 15 *Pseudomonas (1 P. alkylphenolica, 1 P. putida, 10 P. stuzeri,* and 4 *P. vranovensis)*, and 11 *Stenotrophomonas maltophilia* isolates (Figure 2; Tables 2 and 3).

**TABLE 3:** Carbapenem‐resistant soil isolates identified and characterized in this study

Closest species identified by BLAST using 16S rRNA gene^a^	Isolate #	Inhibition zone (diameter in mm)^b^						Carbapenemase^c^
		MP	IM	CF	CI	GE	TE	
*Achromobacter marplatensis*	S10‐1	14	33	14	30	21	27	−
*Bradyrhizobium elkanii*	S11‐1	0	0	0	0	0	0	+
*Cupriavidus alkaliphilus*	S2‐2	0	37	47	43	13	33	−
*Cupriavidus alkaliphilus*	S2‐3	0	37	47	40	13	34	−
*Cupriavidus alkaliphilus*	S2‐4	0	38	45	39	14	32	−
*Cupriavidus respiraculi*	S7‐6	10	18	37	41	14	33	−
*Cupriavidus respiraculi*	S8‐1	8	26	25	39	23	31	−
*Cupriavidus respiraculi*	S8‐2	10	26	29	37	27	29	−
*Cupriavidus respiraculi*	S9‐1	12	17	37	39	20	30	−
*Cupriavidus respiraculi*	S9‐2	13	22	19	39	21	31	−
*Enterococcus durans*	S12‐1	11	20	0	24	14	35	−
*Enterococcus gallinarum*	S7‐2	0	51	17	31	27	35	+
*Enterococcus gallinarum*	S11‐3	15	21	0	21	18	19	−
*Planomicrobium glaciei*	S7‐3	0	0	15	34	0	14	−
*Pseudomonas alkylphenolica*	S2‐1	0	36	45	40	14	33	−
*Pseudomonas putida*	S11‐2	12	35	16	38	0	0	+
*Pseudomonas stutzeri*	S4‐1	14	23	13	41	28	34	−
*Pseudomonas stutzeri*	S4‐2	16	29	17	40	28	32	−
*Pseudomonas stutzeri*	S4‐3	10	24	13	42	34	29	−
*Pseudomonas stutzeri*	S5‐1	16	21	20	39	27	30	−
*Pseudomonas stutzeri*	S5‐2	15	21	19	42	31	31	−
*Pseudomonas stutzeri*	S5‐3	17	20	17	41	33	32	−
*Pseudomonas stutzeri*	S5‐4	14	21	17	39	30	32	−
*Pseudomonas stutzeri*	S5‐5	16	22	17	41	32	32	−
*Pseudomonas stutzeri*	S5‐6	17	23	19	43	32	19	−
*Pseudomonas vranovensis*	S3‐1	10	29	0	26	26	22	+
*Pseudomonas vranovensis*	S3‐2	9	26	21	29	27	19	+
*Pseudomonas vranovensis*	S3‐3	11	30	0	28	26	16	−
*Pseudomonas vranovensis*	S3‐4	11	27	0	35	23	24	−
*Stenotrophomonas maltophilia*	S1‐1	0	0	13	26	11	13	+
*Stenotrophomonas maltophilia*	S1‐2	0	0	12	28	12	14	+
*Stenotrophomonas maltophilia*	S1‐3‐1	0	0	20	23	10	14	+
*Stenotrophomonas maltophilia*	S1‐3‐2	0	0	17	24	11	15	+
*Stenotrophomonas maltophilia*	S1‐4	0	0	18	26	0	13	+
*Stenotrophomonas maltophilia*	S1‐5	0	0	12	25	10	12	+
*Stenotrophomonas maltophilia*	S1‐6	0	0	12	26	10	13	+
*Stenotrophomonas maltophilia*	S1‐7	0	0	13	27	15	15	+
*Stenotrophomonas maltophilia*	S7‐1	0	0	9	27	30	20	+
*Stenotrophomonas maltophilia*	S11‐4	0	0	0	24	0	13	+
*Stenotrophomonas maltophilia*	S11‐5	0	0	0	23	16	11	+

Abbreviations: CF, cefotaxime; CI, ciprofloxacin; GE, gentamicin; IM, imipenem; MP, meropenem; TE, tetracycline.

^a^ For each isolate, we obtained their 16S rRNA gene sequence and used BLAST (Altschul et al., 1997) to determine the closest known strain. In all cases, the DNA identity between our isolate and the top BLAST known strain hit was ≥98% (≥99% for 34 out of 40 isolates).

^b^ To determine whether our isolates were resistant (highlighted in red), intermediate (highlighted in yellow) or sensitive (no highlight) to the antibiotics tested, we used the CSLI zone diameter clinical breakpoint values (Clinical & Laboratory Standards Institute, 2018). For taxa in which the CLSI zone diameter breakpoint values were not available, we used the Enterobacteriaceae values. Enterococci are considered clinically resistant to aminoglycosides even if they test as susceptible in vitro (Clinical & Laboratory Standards Institute, 2018).

^c^ All carbapenemase‐producing isolates were carbapenemase‐positive when the CarbaNP test was performed measuring the hydrolysis of both meropenem and imipenem, and all were confirmed as positives using the mCIM test*.* Carbapenemase production was inducible on all carbapenemase‐producing isolates except for *S. maltophilia* isolates S1‐2 and S1‐3‐2.

Interestingly, the majority of the urban soil isolates belonged to the genera *Pseudomonas* and *Stenotrophomonas*, whereas the most represented agricultural soil isolates belonged to the genus *Cupriavidus* (Figure 2). Overall, we identified carbapenem‐resistant (CR) *Pseudomonas* in 5 (all urban soils) out the 11 samples analyzed; CR *Stenotrophomonas maltophilia* in 3 samples (2 urban and 1 agricultural soil); CR *Cupriavidus* in 1 urban and 3 agricultural soil samples; and CR *Enterococcus* in 3 samples (2 agricultural and 1 urban soil), whereas CR *Achromobacter marplatensis*, *Bradyrhizobium elkanii,* and *Planomicrobium glaciei* were identified only in one agricultural, urban, and agricultural soil samples, respectively (Figure 2; Table 2).

### 3.2 Characterization of the antibiotic susceptibility profile of CRB isolates

**FIGURE 3:**
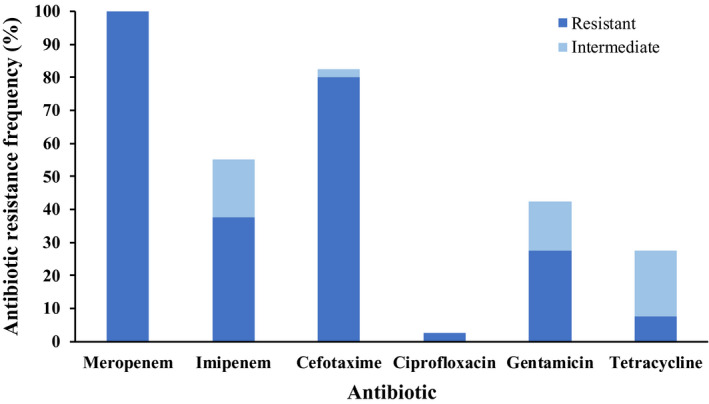
Antibiotic resistance frequency of the soil isolates characterized in this study for carbapenem (meropenem and imipenem) and noncarbapenem (cefotaxime, ciprofloxacin, gentamicin, and tetracycline) antibiotics. For each antibiotic tested, the percentage of resistant isolates is shown in dark blue, and the percentage of intermediate isolates is shown in light blue

We next characterized the antibiotic susceptibility profile of the 40 identified CRB isolates using disk diffusion experiments with the two most clinically used carbapenems (meropenem and imipenem) and 4 noncarbapenem antibiotics (cefotaxime, ciprofloxacin, gentamicin, and tetracycline; Tables 2 and 3; and Figure 3). All 40 isolates were resistant to meropenem, confirming them as CRB. Moreover, most of the isolates were also resistant or intermediate to imipenem (55% of the isolates) and cefotaxime (83% of isolates), which although not a carbapenem, it is also a β‐lactam (third‐generation cephalosporin; Figure 3; Table 3). In contrast, the number of isolates that were resistant to the three different classes of non‐β‐lactam antibiotics tested was much lower. Overall, 43% and 28% of the CRB isolates characterized were resistant or intermediate to aminoglycoside gentamicin and tetracycline, respectively (Figure 3; Table 3). Furthermore, only one CRB isolate, identified as *Bradyrhizobium elkanii*, was resistant to the fluoroquinolone ciprofloxacin (Figure 3; Table 3). These findings highlight the importance of Southern California soils as reservoirs of CRB, including CRB that are also resistant to other antibiotics.

### 3.3 Identification of CRB isolates that produce carbapenemases

Given the importance of carbapenemase genes in spreading resistance to carbapenems, we next used the CarbaNP test to determine which CRB isolates produce carbapenemases. Interestingly, 16 out of the 40 CRB isolates tested (40%) were positive for carbapenemase production when tested by the CarbaNP using both meropenem and imipenem, and as confirmed by the mCIM test (Tables 2 and 3). These carbapenemase‐positive isolates were 1 *Bradyrhizobium elkanii*, *1 E. gallinarum*, 1 *P. putida*, 2 *P. vranovensis*, and all 11 *S. maltophilia* (Table 3). To our knowledge, this is the first report of carbapenemase production for *E. gallinarum* and *P. vranovensis* as well as in the genus *Bradyrhizobium.*

## 4 DISCUSSION

Carbapenem‐resistant bacteria are a major public health threat all over the world (Centers for Disease Control & Prevention, 2013a, 2013b; Cuzon et al., 2011; Guh et al., 2015). However, little is still known about the distribution and characteristics of CRB outside health care or immediately related settings (Gupta et al., 2011; Kallen et al., 2010; Khuntayaporn et al., 2012; Rhomberg & Jones, 2009; Ssekatawa et al., 2018). This gap in knowledge is especially significant in the United States, where only three specific studies about the prevalence of CRB in the environment, all three in freshwater, have been performed (Ash et al., 2002; Aubron et al., 2005; Harmon et al., 2019). CRB in the United States have also been found in fecal samples from dairy farms in New Mexico and Texas and a swine nursery in Ohio (Mollenkopf et al., 2017; Webb et al., 2016). Thus, not only clinical facilities but also farms may contribute to spread CRB to the environment. Recent findings in other parts of the world, especially in Europe, have found CRB in agricultural and nonagricultural soil samples (Borowiak et al., 2017; Djenadi et al., 2018; Fischer et al., 2013; Gudeta et al., 2016; Hrenovic et al., 2019) and suggest that soil may be an underrecognized reservoir of CRB. In the United States, 3 CRB isolates were identified among a collection of penicillin‐resistant isolates obtained from soil samples from the Midwestern United States (Crofts et al., 2018). However, studies that specifically address the distribution and characteristics of CRB in United States soils are still lacking.

The study reported here is significant for several reasons. To our knowledge, this is the first specific study about the distribution and characteristics of CRB in urban and agricultural environmental soils in the United States. Moreover, this is only the second study about the environmental distribution of CRB in the West Coast of the United States, the first one being our previous report on CRB in freshwater (Harmon et al., 2019). Here, we have found that CRB were present in all soil samples analyzed from both from urban and agricultural‐related locations in the Los Angeles–Southern California area. Furthermore, 40% of the CRB isolates characterized were carbapenemase producers (CR‐CRB), and all CRB isolates characterized were resistant or intermediate to at least one of the noncarbapenem antibiotics tested. For most urban soil samples as well as S12 (a hiking trail sample), the relative frequency of CRB compared to the total bacterial counts obtained was less than 1%, which is similar to the relative frequencies of CRB we had previously observed in freshwater environments from the Los Angeles–Southern California area (Harmon et al., 2019). In contrast, most agricultural soil samples (and the urban chicken coop soil samples) had a much higher relative frequency of CRB to the total bacterial count (from 18% up to 80% in soil S7, which was obtained adjacent to a produce farm). Although further studies comparing soil samples from locations at different proximities from farms are necessary, our results support the hypothesis that the use of antibiotics (or the use of manure from antibiotic‐treated animals) in farms might contribute to the spread of CRB to the environment (Mollenkopf et al., 2017; Webb et al., 2016), including CP‐CRB and CRB also resistant to other antibiotics.

In a previous study, Hrenovic et al. (2019) used a similar approach than the one we used in our study, but a different growth medium (CHROMagar™ Acinetobacter medium with CR102 supplement in their study, compared to MacConkey agar medium supplement with meropenem in our study) and temperature (37°C and 42°C in their study, compared to 37°C in our study) to determine the presence of CRB in different soils samples from Croatia. Hrenovic et al. (2019) found that at 37°C, most soil isolates were *S. maltophilia*, except for two soil samples in which they were absent. As is further discussed below, *S. maltophilia* are widespread in soil and other environments, and are intrinsically resistant to carbapenems (Brooke, 2012; Harmon et al., 2019; Tacão et al., 2015; Youenou et al., 2015). They also found that isolating CRB at 42°C, which suppresses the growth of *S. maltophilia*, increased the diversity of CRB recovered from their samples, including CRB of potential anthropogenic origin (Hrenovic et al., 2019). In the future, as we expand our studies to additional soil samples and locations, it will be interesting to analyze our samples at both 37°C and 42°C to compare the abundance and diversity of CRB obtained at both temperatures. However, of the 40 CRB isolates identified and characterized in the present study, only 11 of them (from 3 different soil samples) were *S. maltophilia* (Tables 2 and 3), and we were able to isolate, among other CRB, carbapenem‐resistant (CR) *Cupriavidus,* and *Pseudomonas* strains, as reported by Hrenovic et al. (2019) at 42°C. These findings suggest that, although *S. maltophilia* may be an important contributor to the abundance and wide distribution of CRB found in the soils we analyzed, other CRB were also an important factor. Moreover, although different soil locations were tested in both studies, our findings, as well as those from Djenadi et al. (2018), suggest that at 37°C, using MacConkey medium instead of CHROMagar might contribute to isolating more diverse CRB, even without using 42°C to suppress the growth of *S. maltophilia*. Also, CR *Pseudomonas* were the most abundant (15 out of 40 CRB identified in our study) CRB we found, compared to only one CR *Pseudomona*s isolate identified by Hrenovic et al. (2019) at 42°C. Although further studies analyzing the same soil samples with both growth media and temperatures are necessary, this finding suggests that isolation of CRB at 42°C may not only suppress the growth of *S. maltophilia*, but also of closely related *Pseudomonas*.

To further characterize the diversity of CRB present in the soils we studied, we identified 40 CRB soil isolates. Identification of these isolates revealed a diversity of species that included *Achromobacter marplatensis*, *Bradyrhizobium elkanii*, *Cupriavidus alkaliphilus*, *Cupriavidus respiraculi*, *Enterococcus durans*, *Enterococcus gallinarum*, *Planomicrobium glaciei*, *Pseudomonas alkylphenolica*, *Pseudomonas putida*, *Pseudomonas stuzeri*, *Pseudomonas vranovensis*, and *Stenotrophomonas maltophilia* (Table 3). Of the soil CRB characterized, *Cupriavidus*, *Pseudomonas*, and *S. maltophilia* isolates were the most abundant and widely distributed in soils from the Los Angeles area. Carbapenem‐resistant (CR) *Pseudomonas* and *S. maltophilia* isolates were also the most abundant CRB in freshwater samples from the same area (Harmon et al., 2019) and have been found before both in clinical settings and in soil, freshwater, animal feces, and other environments (Aubron et al., 2005; Brooke, 2012; Centers for Disease Control & Prevention, 2013b; Djenadi et al., 2018; Gudeta et al., 2016; Hrenovic et al., 2019; Tacão et al., 2015; Webb et al., 2016). However, this is to our knowledge the first report of carbapenem‐resistant *P. alkylphenolica* and *P. vranovensis* isolates. Resistance to carbapenems in *Pseudomonas* can occur by different mechanisms such as the production of different carbapenemases, overexpression of efflux pumps, and decreased outer membrane permeability (Papp‐Wallace et al., 2011; Rizek et al., 2014; Rodríguez‐Martínez et al., 2009). Interestingly, only 1 *P. putida* and 2 *P. vranovensis* out of the 15 *Pseudomonas* isolates characterized produced carbapenemases. In contrast, all *S. maltophila* isolates were carbapenemase producers. It is well‐documented that carbapenem resistance in *S. maltophilia* is predominantly caused by the *bla*_L1_ gene, which encodes for the intrinsic L1 carbapenemase in both clinical and environmental isolates (Brooke, 2012; Harmon et al., 2019; Tacão et al., 2015; Youenou et al., 2015). Using PCR, we could confirm that this carbapenemase gene was also present in all our *S. maltophilia* isolates (data not shown).

The third most abundant CR soil isolates obtained belonged to the genus *Cupriavidus*, which we identified in four different samples. Members of this genus are usually found in soil or water environments and occasionally as opportunistic pathogens (Coenye et al., 1999; Coenye, Goris, Spilker, Vandamme, & LiPuma, 2002; Harmon et al., 2019; Henriques et al., 2012; Hrenovic et al., 2019; Karafin et al., 2010; Kobayashi et al., 2016; Wang et al., 2015). However, carbapenem‐resistant *C. alkaliphilus* isolates have not been reported before in either clinical or environmental samples. None of the soil CR *Cupriavidus* isolates characterized in this study produced carbapenemases. A related species, *C. gilardii*, is intrinsically resistant to carbapenems despite also not producing carbapenemases likely because of its large array of multidrug efflux pumps (Ruiz, McCarley, Espejo, Cooper, & Harmon, 2019).

Other notable but less abundant CR soil isolates included *Enterococcus gallinarum*, which is associated with nosocomial‐ and community‐acquired bacteremia and other infections (Narciso‐Schiavon et al., 2015; Quinones, Goni, Rubio, Duran, & Gomez‐Lus, 2005; Reid, Cockerill, & Patel, 2001; Schouten, Voss, & Hoogkamp‐Korstanje, 1999); *Enterococcus durans*, an infrequent human pathogen mostly associated with diarrhea in piglets and calves (Cheon & Chae, 1996; Quinones et al., 2005; Rogers, Zeman, & Erickson, 1992; Schouten et al., 1999); *Achromobacter marplatensis,* a soil microbe that has also been found in cystic fibrosis patients (Gomila et al., 2011; Papalia et al., 2019); *Bradyrhizobium elkanii,* a soil bacterium and legume symbiont used commercially as an inoculant to improve the growth of legume plants (Crovadore et al., 2016; Faruque et al., 2015; Hungria, Delamuta, Ribeiro, & Nogueira, 2019); and *Planomicrobium glaciei*, an infrequently isolated bacterium, first found in a glacier and later in food (Tshipamba, Lubanza, Adetunji, & Mwanza, 2018; Zhang et al., 2009). Finding these isolates is significant for several reasons. First, neither resistance to carbapenems in these species nor the production of carbapenemases found in one *E. gallinarum* isolate and the *B. elkanii* isolate has been reported before. Given that *E. gallinarum* can cause infections in humans, including bacteremia, resistance to carbapenems and carbapenemase production in this species may impact therapy directly or by the transmission of the carbapenemase gene to other pathogens. In the case of *B. elkanii*, although it is not known to infect humans or animals, the fact that this isolate was completely resistant (0 mm inhibition zone diameters) to all carbapenem and noncarbapenem antibiotics tested makes this bacterium a potential reservoir of multiple antibiotic resistance genes. Further genomic studies are necessary to fully characterize this isolate and determine whether its antibiotic resistance determinants are conserved among other *B. elkanii* isolates and whether these determinants are located in mobile elements that may facilitate their transmission to other bacteria. However, the role of this species as a potential reservoir of resistance genes should be taken into account when considering its commercial use in crops.

## 5 CONCLUSIONS

In conclusion, our findings show for the first time that soils from the Los Angeles–Southern California area are a previously underappreciated reservoir of different species of CRB that are also resistant to other antibiotics, including carbapenemase‐producing CRB. Our study also shows a much higher relative frequency of CRB on most soils from locations adjacent to farms, compared to most soils from urban locations, which suggest a potential role of farms in spreading bacteria resistant to carbapenems and other antibiotics.

## CONFLICTS OF INTEREST

None declared.

## AUTHOR CONTRIBUTION

**Nicolas V. Lopez:** Conceptualization (supporting); Data curation (lead); Formal analysis (equal); Investigation (lead); Methodology (equal). **Cameron J. Farsar:** Data curation (supporting); Formal analysis (supporting); Investigation (supporting). **Dana E. Harmon:** Conceptualization (supporting); Data curation (supporting); Formal analysis (supporting); Investigation (supporting); Methodology (supporting); Supervision (supporting). **Cristian Ruiz:** Conceptualization (lead); Data curation (supporting); Formal analysis (equal); Funding acquisition (lead); Investigation (supporting); Methodology (equal); Project administration (lead); Supervision (lead); Writing‐original draft (equal); Writing‐review & editing (lead). 

## ETHICS STATEMENT

None required.

## Data Availability

All 16S rRNA gene sequences obtained in this study have been deposited in GenBank (https://www.ncbi.nlm.nih.gov/genbank/) under the following accession numbers: MN732973–MN733008, MN810328–MN810330, and MN813762.
